# Assessing *Campylobacter* Colonization of Broiler Herds Ante Mortem and Monitoring *Campylobacter* Contamination Post Mortem by qPCR

**DOI:** 10.3390/pathogens9090742

**Published:** 2020-09-10

**Authors:** Gudrun Overesch, Katrin Haas, Peter Kuhnert

**Affiliations:** Institute of Veterinary Bacteriology, University of Bern, 3012 Bern, Switzerland; katrin.haas@bluewin.ch (K.H.); peter.kuhnert@vetsuisse.unibe.ch (P.K.)

**Keywords:** *Campylobacter jejuni*, *Campylobacter coli*, process hygiene criterion, chicken meat, enumeration, campylobacteriosis, slaughterhouse, carcasses

## Abstract

Human campylobacteriosis is the most prevalent zoonosis, with chicken meat contributing substantially to the number of cases. Measures to avoid or at least reduce exposure by meat contaminated with *Campylobacter* (*C*.) spp. are needed. With regard to the process hygiene criterion introduced in 2018 for *Campylobacter* spp. on broiler carcasses, we evaluated the performance of a recently developed quantitative real-time PCR (qPCR) for *C. jejuni/coli* on random caecal samples and chicken meat. With the qPCR on pooled caecal samples not only *C. jejuni/coli* positive (69.6%) versus negative broiler herds (30.4%) were identified, but herds highly colonized with *C. jejuni/coli* (39.4%) could also be identified. From the chicken meat samples, 8.0% were positive for *C. jejuni/coli* by qPCR and 0.7% by enumeration (>10 cfu/g) compared to 58.3% using cultural enrichment. Given the higher sensitivity, the qPCR method could replace the currently used enumeration method to assess the process hygiene criterion for *Campylobacter* spp. on broiler carcasses. Moreover, with the qPCR, a reliable identification of *C. jejuni/coli* colonized incoming broiler herds a few days before slaughter is feasible, which provides important information to optimize slaughter processes. Finally, identifying and monitoring herds with high *C. jejuni/coli* colonization rates could help to individually improve biosecurity measures at farm level, eventually reducing the *C. jejuni/coli* load on chicken meat.

## 1. Introduction

For more than 15 years, human campylobacteriosis has been a major food-borne disease in Europe with more than 240,000 reported cases per year [1]. The extraordinary high socio-economic burden is substantiated by the fact that the notification rate corresponds to an estimated number of about nine million cases of human campylobacteriosis annually in Europe [2]. The disease is mainly caused by *Campylobacter* (*C.*) *jejuni* and to a lesser extent by *C. coli* with approx. 84% and 10% of cases, respectively [1]. The handling, preparation, and consumption of chicken meat is a major risk factor and up to 50–80% of human cases could be attributed to *C. jejuni/coli* isolates from the chicken reservoir as a whole [3,4]. A baseline study in 2008 showed that, within the European Union, on average, 75.8% of broiler carcasses at slaughterhouse are contaminated with *Campylobacter* spp. [5]. In 2018, broiler meat is still the fresh meat category with the highest contamination rates with *C. jejuni/coli* (37.5%) and 26.0% of tested broiler herds were positive for *C. jejuni/coli* [1]. *C. jejuni/coli* are commensal bacteria in the gut microbiota of broilers and colonized animals do not show any signs of disease when getting to slaughter [6]. Incoming *C. jejuni/coli* positive herds result in *C. jejuni/coli* positive carcasses and via cross contamination of the processing line in the slaughterhouse, even broiler carcasses of negative herds may become positive at the end of the slaughter process [7]. Since the cooling step is not able to totally inactivate *C. jejuni/coli*, the organism remains on broiler meat up to the retail level and thus reaches the consumer [8].

In Switzerland, notified human campylobacteriosis cases slightly decreased from 7688 in 2016 to 7223 confirmed cases in 2019, this corresponds to a decrease in incidence of 92 to 84 cases per 100.000 inhabitants [9,10]. Most cases were caused by *C. jejuni* (2019: 68% of all cases, in 24% of cases no distinction was made between *C. jejuni* and *C. coli*). The typical summer peak occurred in the months of July and August accounting for 1817 cases in 2019. The Swiss poultry meat industry conducts annually approximately 1400 *Campylobacter* examinations on carcasses and meat from broiler. About 21.8% of the samples proved to be positive for *Campylobacter* spp. in 2019.

In 2018, for the first time, regulations concerning *Campylobacter* spp. were introduced into the European legislation on food safety and hygiene (Commission Regulation (EU) no 2017/1495 on amending regulation EC no 2073/2005 as regards *Campylobacter* in broiler carcasses). The new process hygiene criterion (PHC) is based on the *Campylobacter* spp. count on broiler carcasses after chilling. According to this criterion, slaughterhouses have to control, and in cases of non-compliance improve, their hygiene management in the slaughter process and/or the biosecurity measures on the broiler farms if 20 (in 2018 and 2019) out of 50 carcasses show more than 1000 cfu/g *Campylobacter* spp. after chilling. This criterion will be tightened in 2020 and 2025 with only 15 and 10 samples allowed to reach this value, respectively. Therefore, identification and control of incoming *Campylobacter* spp. positive broiler herds before slaughter will get more critically important for slaughterhouses in the future to comply with the European regulation.

With the introduction of the PHC, reliable quantification of the *Campylobacter* spp. load is of major importance for decision makers, as non-compliance will lead to cost-intensive measures to be taken. The prescribed method given by to legislation is the enumeration according to EN ISO 10272-2:2017. This method is laborious and time consuming, leading to the loss of valuable time before the realization of improved hygiene and biosecurity measures in the production process could take place. Hence, sensitive, reliable, and faster methods in comparison to the enumeration technique are needed. A quantitative real-time PCR (qPCR) that allows an easy, sensitive, and quantitative method for *C. jejuni/coli* detection in poultry flocks ante mortem by the analysis of faecal, caecal, and boot sock samples was published recently [11]. The technique’s efficiency was 93% and it showed a broad range of linearity down to the detection limit of two genome equivalents in the reaction. There was good correlation between Ct mean values and cfu/g (correlation coefficient = 0.8732, *p* < 0.0001) in all samples, whether they contained high or low numbers of *C. jejuni/coli*. At the farm level, it could be shown that not only caecal content as the gold standard, but also boot sock samples, analyzed by qPCR is an efficient way to detect and quantify *C. jejuni/coli* in broiler herds a few days before slaughter. Thereby a sensitive and standardized classification system into negative, low, moderate, or high *C. jejuni/coli* colonization of broiler herds ante mortem is available.

The aim of this study was to evaluate the qPCR as a tool for systematic monitoring of broiler herds on a random sample over one year to get new insights into the quantitative *C. jejuni/coli* load and possible differences between broiler herds. Moreover, the qPCR protocol should be evaluated for its application on chicken meat as an alternative to the method for culture-based enumeration of *Campylobacter* spp. according to EN ISO 10272-2:2017.

## 2. Results

### 2.1. Classification of C. jejuni/coli Colonization of Broiler Herds by qPCR

**Figure 1:**
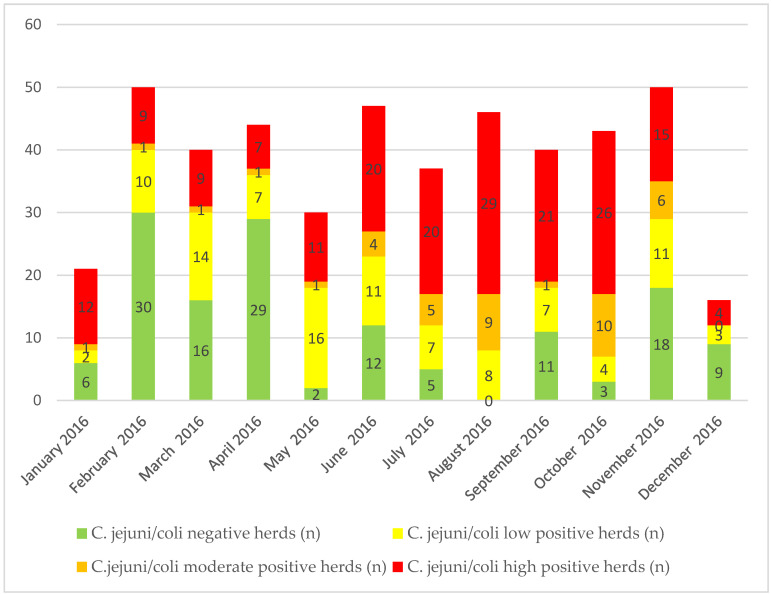
*C. jejuni/coli* colonization status of pooled caecal samples from broiler herds by qPCR in relation to the sampling date. Numbers in bars indicate number of positive herds. *C. jejuni/coli* negative herds: Ct value ≥ 40; *C. jejuni/coli* low positive herds: Ct value < 40 to ≥ 35; *C. jejuni/coli* moderate positive herds: Ct value < 35 to ≥ 30; *C. jejuni/coli* high positive herds: Ct value < 30.

From a total of 484 pooled caecal samples (herds) from 464 (95.9%) a reliable qPCR result was obtained while in 20 samples the qPCR was inhibited. Nine of the inhibited samples were taken in May, three in June, two in July, and six in November. A total of 141 herds (30.4%; 26.4–34.7, 95%CI) were negative for *C. jejuni/coli*. One hundred herds turned out to be colonized at low level (21.6%; 18.1–25.5, 95%CI), 40 herds were moderately colonized (8.6%; 6.4–11.5 95%CI) and 183 were identified as highly colonized herds (39.4%; 35.1–44.0, 95%CI) (Figure 1).

The highest numbers of *C. jejuni/coli* negative herds were observed in February and April, with a decrease until August (Figure 1). In autumn, the number of negative flocks increased again, but at a lower level. The highest numbers of highly positive herds were observed from June to October, with a peak in August. Interestingly, besides this seasonal trend, *C. jejuni/coli* positive as well as negative herds were observed throughout the year.

When comparing the detection rates of *C. jejuni/coli* positive herds per slaughterhouse the proportion of *C. jejuni/coli* low to high positive herds were found to be lower in the two biggest slaughterhouses 1 and 2 (66.3%; 59.4–72.6, 95%CI and 66.3%; 58.6–73.1, 95%CI) than in the smaller slaughterhouses 3 and 4 (79.4%; 67.8–87.5, 95%CI and 79.6%; 65.5–88.9, 95%CI) (Table 1). This trend is even more pronounced with the proportion of *C. jejuni/coli* high positive herds, which was much lower in the two biggest slaughterhouses 1 and 2 (27.1%; 21.5–33.5, 95%CI and 37.8%; 30.7–45.4, 95%CI) than in the smaller slaughterhouses 3 and 4 (61.5%; 49.4–72.4, 95%CI and 50.0%; 35.8–64.2, 95%CI) (Table 1).

### 2.2. Comparison of C. jejuni/coli Colonization of Broiler Herds by qPCR and by Direct Culture Detection

For all 484 pooled caecal samples analyzed by qPCR, direct culture detection of *C. jejuni/coli* onto mCCDA was also performed. For 303 samples (62.6%; 58.2–66.8, 95%CI), no *C. jejuni/coli* could be detected while 161 samples (33.3%; 29.2–37.6, 95%CI) were positive for *C. jejuni/coli* (Table 2). All samples negative for *C. jejuni/coli* by qPCR were also negative by culture. From 97 low positive samples by qPCR, only three were positive by culture and from 38 moderate positive samples by qPCR only in two samples *C. jejuni/coli* could be detected by culture. From the 156 qPCR high positive samples, 27 turned out to be negative by direct culture detection.

### 2.3. Classification of C. jejuni/coli Contamination of Fresh Chicken Meat by qPCR

Between April 2016 and March 2017, a total of 281 fresh retail chicken meat samples were analyzed by qPCR. For 19 samples the qPCR was inhibited (one each in July, October and November, two in September, three in August, 11 samples in May; 15 originated from Switzerland, 4 from other countries). Hence, for 262 samples a qPCR result was obtained (Table 3). In total, 241 out of the 262 samples tested negative for *C. jejuni/coli* by qPCR (92.0%; 88.1–94.7, 95%CI). Twenty-one samples were low positive (8.0%; 5.3–11.9, 95%CI) and distributed over a period from August 2016 until March 2017. According to the sampling plan (two in three samples from Switzerland) from 281 chicken meat samples 194 (69%) were domestic and 87 (31%) originated from foreign countries. Independent of their origin the same proportion of *C. jejuni/coli* negative and positive samples was observed (Table 3).

### 2.4. Comparison of the C. jejuni/coli Contamination of Fresh Chicken Meat by qPCR and by Enumeration

From the 281 fresh chicken meat samples which were analyzed by qPCR, enumeration of *C. jejuni/coli* was performed in parallel (Table 4). For 19 samples which were inhibited by qPCR, all were negative for *C. jejuni/coli* by enumeration (<10 cfu/g). Only two samples which showed a negative qPCR result tested positive for *C. jejuni/coli* by enumeration (>10 cfu/g) (0.7 %; 0.2–2.6, 95%CI) (Table 4). From 239 samples tested negative by enumeration, 21 turned out low positive in the qPCR.

### 2.5. Comparison of C. jejuni/coli Contamination of Fresh Chicken Meat by Enrichment and Enumeration

The 211 fresh chicken meat samples were also analyzed for *C. jejuni/coli* by enrichment of 25 g in Preston broth and enumeration in parallel (Table 5). In total, 123 out of the 211 samples tested positive for *C. jejuni/coli* by Preston enrichment (58.3%; 51.6–64.7, 95%CI). In contrast, only two samples were positive for *C. jejuni/coli* by enumeration (71 cfu/g and 13 cfu/g) (0.95%; 0.3–3.4, 95%CI) (Table 5). These two samples were positive with enrichment, too.

## 3. Discussion

A key factor for a substantial reduction of *C. jejuni/coli* positive broiler carcasses at slaughterhouse is the colonization level of incoming herds which affects the entire slaughter process eventually resulting in corresponding *C. jejuni/coli* counts on carcasses [12]. In our study, only 30.4% of the broiler herds were tested negative for *C. jejuni/coli* by qPCR and 69.6% were positive, of which a remarkable proportion (39.4%) were highly positive (Ct value <30) for *C. jejuni/coli*. Previous studies have shown, that a Ct value of 30 corresponds roughly to 1 × 10^5^ (10^4^ to 10^6^) cfu/g *C. jejuni/coli* depending on the matrix tested [11,13]. Seasonal trends on *C. jejuni/coli* prevalence were observed, which follows in general the trends in human campylobacteriosis, with a prominent peak in summer and a second peak around new year eve [1,14]. However, critically important, highly positive herds occurred not only during summer, but also in autumn and winter. Besides the seasonal effect, *Campylobacter* colonization of broiler flocks varies—at least in Europe—between countries as well, with Norway, Sweden, and Finland showing the lowest prevalence [1]. This raises the question about the differences in management and biosecurity measures between farms with constantly no or low level and high level of intestinally colonized broilers. The colonization of broiler intestine with *C. jejuni/coli* depends on several factors, including breed, animal density, special diet, or access to the outside. [15,16,17]. Moreover, individual farm characteristics, such as poor biosecurity (e.g., inadequate hygiene measures, access of vectors), the presence of other animal species on the farm, catching and placing measures and management of crates have been shown to increase *C. jejuni/coli* colonization and excretion rates of broilers [18,19,20,21]. With the semi-quantitative approach of our qPCR the most critical, high *C. jejuni/coli* shedding farms could be identified ante mortem and decision makers could primarily focus on these farms for analyzing and improving biosecurity measures. In Switzerland, a great proportion of broilers are produced on farms with outdoor access, to meet expectations of the consumer for more animal welfare-friendly rearing systems. Since we had no information on the rearing system, future studies could base on our data and investigate, if such farms possibly account for the big proportion of highly positive *C. jejuni/coli* animals. Interestingly, we could observe, that the two biggest slaughterhouses supplied caceal samples with an overall lower proportion of *C. jejuni/coli* positive qPCR results than the two smaller slaughterhouses. This may be due to differences in the farm biosecurity systems, which deliver the broilers. On the other hand, this could point out to different hygiene measures at slaughterhouse when taken the samples. Further studies are needed to prove the underlying responsible factors for this observation. Overall, determination of the incoming *C. jejuni/coli* load of flocks ante mortem, either in caeca or using boot socks, will help decision makers to identify and optimize the whole process along the farm to fork production line. Recently, EFSA has given an updated review of the most effective control options [22]. Besides some uncertainties while modelling, control options like vaccination, feed and water additives, and discontinued thinning turned out to lower the risk for *C. jejuni/coli* load in broiler caeca. The potential of such an approach was recently shown by Frosth et al. [23], where the prevalence of Swedish *Campylobacter* spp. positive flocks could be lowered from 15.4% in 2016 to 4.6% in 2019.

Caecal samples were analyzed by direct culture detection for *C. jejuni/coli* in parallel. The prevalence of positive herds (33.3%) by direct detection was more than twice as low than the prevalence by qPCR (69.6%). Full agreement between both methods was achieved for the negative tested samples (*n* = 141). From the 183 samples tested highly positive for *C. jejuni/coli* by qPCR, the vast majority (*n* = 156, 85.3%) was also positive by culture. With 27 samples being highly positive by qPCR the detection of *C. jejuni/coli* by culture was not possible. The most likely explanation for this is the occurrence of dead or viable but non-culturable (VBNC) *C. jejuni/coli* [24]. One could argue that the detection of dead or VBNC *C. jejuni/coli* is not relevant when focusing on human campylobacteriosis. However, the detection of VBNC or even dead *C. jejuni/coli* indicates problems with biosecurity measures on farm or slaughterhouse level. Moreover, VBNC might still be capable to induce disease in humans [25]. In nearly all of the samples categorized as low (*n* = 100, 97.0%) and moderate positive (*n* = 40, 95.0%) by qPCR, the cultural detection failed. This is in line with other studies demonstrating the higher sensitivity of PCR protocols versus culture methods with or without enrichment for *C. jejuni/coli* in different matrices [25,26]. Together with the previously shown highly reliable quantification by qPCR [11], our study corroborated the finding that qPCR turned out to be much more sensitive than the direct detection, not to mention its speed, robustness, and shorter handling time. For decision makers, it is crucial to be aware that the estimated *C. jejuni/coli* prevalence in Swiss broiler herds determined by the currently used culture based standard method is highly underestimated, especially for herds with low and moderate colonization.

The recently introduced PHC focused on broiler carcasses that are contaminated with more than 1000 cfu/g *Campylobacter* spp. as determined by the enumeration method according to EN ISO 10272-2:2017. Therefore, we applied the established qPCR on fresh chicken meat samples as approximation for carcasses in order to evaluate the potential for monitoring *C. jejuni/coli* contamination at slaughterhouses in comparison to the prescribed cultural enumeration method. It turned out that only two out of 262 chicken meat samples showed >10 *C. jejuni/coli* cfu/g (0.8%). These low level contaminated samples (71 cfu/g and 13 cfu/g) were tested negative by qPCR. Such a discrepancy was not observed with caecal content. We assume that inhomogeneous distribution of bacteria in the initial peptone water suspension together with the small amount of 250 µL used for the qPCR might be the reason for the negative qPCR result. On the other hand, 21 out of 262 samples (8.0%) were tested low positive for *C. jejuni/coli* by qPCR only. Based on this performance one can assume that discrepancies between qPCR results in enriched broth compared to results by cultural enrichment would be less pronounced. This result may reflect sufficient sensitivity of the qPCR protocol, but further validation studies on broiler carcasses should be performed to be able to replace the cultural enumeration by qPCR for determination of PHC in the future.

The prevalence and contamination level of fresh chicken meat determined by enumeration (0.7%) as well as by qPCR (8.0%) seems to be very low, even before the PHC was introduced in 2018. When interpreting these results, one has to take into account that predominantly sliced breast meat without skin was tested, which is known to be less contaminated than meat with skin. However, this finding is somewhat challenged when looking at the results for detection of *C. jejuni/coli* by enrichment according to EN ISO 10272-1:2017. The prevalence of *C. jejuni/coli* contamination in fresh chicken meat analyzed by enrichment was very high (58.3%) compared to direct culture and qPCR. This may partially be due to the detection limit of 10 cfu/g with enumeration. However, since the infection dose for human camplyobacteriosis is very low (approx. 550 cfu) [27,28] application of the PHC established now for broiler carcasses at slaughterhouses after chilling might highly underestimate the percentage of relevant *C. jejuni/coli* contaminated broiler carcasses, especially in countries with a low percentage of highly contaminated broiler carcasses (>1000 cfu/g) like Finland, Sweden, or Norway [5]. EFSA estimated that the risk for human campylobacteriosis in Europe will be reduced by >50% if no slaughter batches achieve the limit of >1000 cfu/g on breast and neck skin [1]. On a country level the success for decreasing the number of human cases is highly correlated with the number of batches, which fall below the currently valid PHC. On the European level first data reported from 2018 indicate that approx. 18% of neck skin samples tested, showed *C. jejuni/coli* numbers >1000 cfu/g [1]. This proportion of heavily contaminated broiler carcasses seems to be low and the development of the reported cases of human campylobacteriosis in Europe will show if it will be effective in lowering substantially the case numbers in the future.

## 4. Materials and Methods

### 4.1. Sampling and Samples Preparation

In 2016, caecal samples were taken from 484 stratified random herds in Swiss poultry slaughterhouses. Caecal samples were collected in the five largest poultry slaughterhouses from 11.01.2016 until 13.12.2016. Every slaughterhouse collected a number of samples proportional to the number of animals slaughtered per year. This procedure ensured that at least 75% of slaughtered broilers were covered by the sample size. Sampling was spread evenly throughout the year. Five randomly chosen caecal samples per broiler herd were taken. Caecal samples were stored at 4–8 °C for a maximum of 48 h before cultivation and at −80 °C until the qPCR analyses were performed. The five ceaca from each herd were opened and the content was mixed with sterile instruments to get the pooled caecal sample.

From January to December 2016, 302 chicken meat samples (min. 50 g) were taken from fresh, skinless, chilled, packed without modified atmosphere, minced and not minced and otherwise untreated meat sold at retail level. Samples were collected in all Swiss cantons throughout the year. The applied sampling scheme considered each canton’s population density and market shares of retailers. Approximately half of the chicken meat consumed in Switzerland is imported. Hence, imported and domestic chicken meat accounted for approximately one third and two thirds respectively.

### 4.2. DNA Extraction and PCR

DNA extraction for qPCR was done from 250 μg of the pooled caecal content using the FastDNA Spin Kit for Soil (MP Biomedicals, Solon, OH, USA) following the manufacturers instruction. For meat samples 250 μL of the incubated buffered peptone water solution containing 10 g chicken meat used for enumeration, was taken for DNA extraction as described above.

Probes and primers for the qPCR as well as the internal inhibition control used were previously described. [11,13]. The reaction mix consisted of 1× TaqMan Fast Advanced Mastermix (Thermo Fisher Scientific, Reinach, Switzerland), 300 nM of each primer Ccj_fusA-L1 (GCCTTGAGGAAATTAAAACTGGTATT), Ccj_fusA-L2 (GCCTTGAAGAGATTAAAACAGGGATT), Ccj_fusA-R1 (TTTAAATGCAGTTCCACAAAGCA), Ccj_fusA-R2 (TTTAAACGCTGTACCGCAAAGCA), 200 nM of each FAM-MGB labeled probe Cj_fusA-probe (AAGTCTTTCTATCGTTCC) and Cc_fusA-probe (AAGTCTTTCTATTGTTCC). The Exogenous Internal Positive Control (IPC; Thermo Fisher Scientific, Reinach, Switzerland) with the IPC mix diluted 1:20 and the IPC template diluted 1:100 was included in every reaction. Each sample was tested in duplicates using 2.5 μL DNA in a total volume of 25 μL. In each run five positive controls with 5650 genome equivalents (GEq)/μL, 565 GEq/μL, 56.5 GEq/μL, 5.65 GEq/μL and 0.565 GEq/μL for creation of the standard curve was included. The samples were run on an ABI 7500 Fast Real-Time PCR System using standard thermal cycling conditions and analyzed with the 7500 software, version 2.0.5 (Thermo Fisher Scientific, Reinach, Switzerland). For all samples, a threshold of 0.02 was set. For each run the standard curve was analyzed for outliers and slope. Standard curves with a slope between −3.58 and −3.10 were used for the analysis. According to the mean Ct value results samples were categorized as *C. jejuni/coli* negative (Ct values ≥ 40), *C. jejuni/coli* low colonized (Ct values < 40 to ≥ 35), *C. jejuni/coli* moderately colonized (Ct values < 35 to ≥ 30) and *C. jejuni/coli* highly colonized (Ct values < 30) as described by Hass et al. [11].

### 4.3. Direct and Pre-Enrichment Culturing

Caecal samples were tested for *C. jejuni/coli* by direct culture detection on modified charcoal cefoperazone deoxycholate agar (mCCDA; Oxoid Ltd., Basingstoke, UK). A loopful of the pooled caecal content was spread onto mCCDA plates. After incubation at 41.5 ± 1 °C for 48 h at microaerobic conditions (5% O_2_, 5% CO_2_, 80% N_2_ and 10% H_2_). Suspicious colonies were transferred onto tryptone soy agar plates containing 5% sheep blood (TSA-SB; BD Becton Dickinson, Franklin Lakes, NJ, USA) at 37 ± 1 °C for 24 h. Identification of suspicious colonies was carried out by matrix-assisted laser desorption/ionization time-of-flight mass spectroscopy (MALDI TOF MS; Bruker Daltonics, Bremen, Germany) using the direct transfer method according to the manufacturer’s recommendations [29].

For enumeration of *C. jejuni/coli* in fresh meat samples the method according to EN ISO 10272-2:2017 was applied. In brief, 10 g of chicken meat was minced with a scissors and homogenized in 90 mL buffered peptone water (Axonlab, Bern, Switzerland) with a stomacher (Stomacher 400 circulator, Seward, UK). One mL of the suspension as well as 100 µL of decimal dilutions of the suspension in sterile 0.9% NaCl was spread out onto mCCDA plates. After incubation, suspicious colonies were counted and up to five colonies were transferred onto TSA-SB agar plates. Identification was performed as described above.

For the enrichment of *C. jejuni/coli* in fresh meat samples, 25 g of minced chicken meat was homogenized in 225 mL Preston broth (Thermo Fisher Scientific, Reinach, Switzerland) with a stomacher. After incubation at 37 ± 1 °C for 4 h and subsequent incubation at 41.5 ± 1 °C for 48 h at microaerobic conditions one loopful of the suspension was spread onto mCCDA plates. After incubation at 41.5 ± 1 °C for 48 h, suspicious colonies were transferred onto TSA-SB agar plates. Identification was performed as described above.

Confidence intervals were calculated using the R (version 3.4.1, www.cran.r-project.org) function binom.test.

## 5. Conclusions

The recently developed qPCR protocol was further evaluated on pooled caecal samples and proved useful for assessing the *C. jejuni/coli* load of broiler batches shortly before slaughter. This provides an exceedingly helpful tool for decision makers to control and to improve hygiene measures at slaughter. Moreover, knowledge of constantly high shedding farms provides the basis to individually advise them in good biosecurity management, thereby reducing prevalence and load of *C. jejuni/coli*. The qPCR is more sensitive for the quantification of the *C. jejuni/coli* contamination rate on carcasses than the prescribed cultural enumeration method and could replace it for determination of the currently introduced PHC in the European legislation. As a summarized conclusion, the qPCR is an important and suitable tool to enable reduction of human exposure to *C. jejuni/coli* contaminated chicken meat, thereby reducing the number of campylobacteriosis cases and improving public health.

## Figures and Tables

**Table 1:** *C. jejuni/coli* colonization status of pooled caecal samples from broilers by qPCR in relation to the slaughterhouse.

	*C. jejuni/coli* Negative Herds ^1^ (*n*)	*C. jejuni/coli* Low Positive Herds ^2^ (*n*)	*C. jejuni/coli* Moderate Positive Herds ^3^ (*n*)	*C. jejuni/coli* High Positive Herds ^4^ (*n*)	Caecal Samples with Inhibited qPCR (*n*)	Total Caecal Samples Per Slaughter-House (*n*)
Slaughter-house 1	65	47	25	56	14	207
Slaughter-house 2	54	36	8	62	4	164
Slaughter-house 3	13	7	3	40	2	65
Slaughter-house 4	9	10	3	22	0	44
Slaughter-house 5	0	0	1	3	0	4
**Total**	**141**	**100**	**40**	**183**	**20**	**484**

^1^ Ct value ≥ 40; ^2^ Ct value < 40 to ≥ 35; ^3^ Ct value < 35 to ≥ 30; ^4^ Ct value < 30.

**Table 2:** Comparison of the *C. jejuni/coli* status of broiler herds by qPCR and by direct culture detection.

Results of Caecal Samples by Direct Detection:	Results of Caecal Samples by qPCR:				
	Number of *C. jejuni/coli* Negative ^1^ Herds (*n*)	Number of *C. jejuni/coli* Low Positive ^2^ Herds (*n*)	Number of *C. jejuni/coli* Moderate Positive ^3^ Herds (*n*)	Number of *C. jejuni/coli* High Positive ^4^ Herds (*n*)	Total
Number of *C. jejuni/coli* negative flocks	141	97	38	27	303
Number of *C. jejuni/coli* positive flocks	0	3	2	156	161
**Total**	**141**	**100**	**40**	**183**	**464**

^1^ Ct value ≥ 40; ^2^ Ct value < 40 to ≥ 35; ^3^ Ct value < 35 to ≥ 30; ^4^ Ct value < 30

**Table 3:** *C. jejuni/coli* classification of chicken meat samples by qPCR in relation to the sampling date.

Sampling Month	*C. jejuni/coli* Negative ^1^ Meat Samples (*n*)	*C. jejuni/coli* Low Positive ^2^ Meat Samples (*n*)	*C. jejuni/coli* Moderate Positive ^3^ Meat Samples (*n*)	*C. jejuni/coli* High Positive ^4^ Meat Samples (*n*)
April 2016	28	0	0	0
May 2016	16	0	0	0
June 2016	18	0	0	0
July 2016	24	0	0	0
August 2016	18	1	0	0
September 2016	25	5	0	0
October 2016	14	4	0	0
November 2016	16	5	0	0
December 2016	12	1	0	0
January 2017	23	2	0	0
February 2017	22	2	0	0
March 2017	25	1	0	0
**Total**	**241**	**21**	**0**	**0**
Swiss origin	164	15	0	0
Other origin	77	6	0	0

^1^ Ct value ≥ 40; ^2^ Ct value < 40 to ≥ 35; ^3^ Ct value < 35 to ≥ 30; ^4^ Ct value < 30.

**Table 4:** Comparison of the *C. jejuni/coli* status of fresh chicken meat by qPCR and by enumeration.

Results by Enumeration	Results by qPCR:				
	Number of *C. jejuni/coli* Negative ^1^ Meat Samples	Number of *C. jejuni/coli* Low Positive ^2^ Meat Samples	Number of *C. jejuni/coli* Moderate Positive ^3^ Meat Samples	Number of *C. jejuni/coli* Highly Positive ^4^ Meat Samples	Total
Number of *C. jejuni/coli* negative meat samples (<10 cfu/g) (*n*)	239	21	0	0	**260**
Number of *C. jejuni/coli* positive meat samples (>10 cfu/g) (*n*)	2	0	0	0	**2**
**Total**	**241**	**21**	**0**	**0**	**262**

^1^ Ct value ≥ 40; ^2^ Ct value < 40 to ≥ 35; ^3^ Ct value < 35 to ≥ 30; ^4^ Ct value < 30.

**Table 5:** *C. jejuni/coli* classification of chicken meat samples by Preston enrichment and by enumeration in relation to the sampling date.

Sampling Month:	*C. jejuni/coli* Negative Meat Samples by Enrichment in 25 g (*n*)	*C. jejuni/coli* Positive Meat Samples by Enrichment in 25 g (*n*)	*C. jejuni/coli* Negative Meat Samples by Enumeration (<10 cfu */g) (*n*)	*C. jejuni/coli* Positive Meat Samples by Enumeration (>10 cfu/g) (*n*)
April 2016	12	16	28	0
May 2016	8	19	27	0
June 2016	11	12	23	0
July 2016	6	19	24	1
August 2016	11	11	21	1
September 2016	14	18	32	0
October 2016	9	10	19	0
November 2016	10	12	22	0
December 2016	7	6	13	0
**Total**	**88**	**123**	**209**	**2**

* colony forming unit.
